# Heterogeneity of G6PD deficiency prevalence in Mozambique: a school-based cross-sectional survey in three different regions

**DOI:** 10.1186/s12936-016-1674-y

**Published:** 2017-01-19

**Authors:** Beatriz Galatas, Lurdes Mabote, Wilson Simone, Gloria Matambisso, Lidia Nhamussua, María del Mar Mañú-Pereira, Clara Menéndez, Francisco Saute, Eusebio Macete, Quique Bassat, Pedro Alonso, Pedro Aide

**Affiliations:** 1 Centro de Investigação em Saúde de Manhiça (CISM), Maputo, Mozambique; 2 ISGlobal, Barcelona Ctr. Int. Health Res. (CRESIB), Hospital Clínic-Universitat de Barcelona, Barcelona, Spain; 3 Hospital Clinic-Universitat de Barcelona, Barcelona, Spain; 4 National Directorate of Health, Ministry of Health, Maputo, Mozambique; 5 ICREA, Pg. Lluís Companys 23, 08010 Barcelona, Spain; 6 National Institute of Health, Ministry of Health, Maputo, Mozambique

**Keywords:** Glucose-6-phosphate-dehydrogenase, Deficiency, Mozambique

## Abstract

**Background:**

Glucose-6-phosphate dehydrogenase (G6PD) deficiency is an X-linked hereditary enzymatic abnormality that affects more than 400 million people worldwide. Most deficient individuals do not manifest any symptoms; however, several precipitant agents—such as fava intake, infections, or several drugs—may trigger acute haemolytic anaemia. Countries should be informed of the prevalence of this enzymatic anomaly within their borders, in order to make safe and appropriate national decisions regarding the use of potentially unsafe drugs for G6PD deficient individuals.

**Methods:**

A school-based cross-sectional survey was conducted in three districts in Mozambique, namely Manhiça, located in the south; Mocuba in the centre; and Pemba in the northern tip of the country. G6PD deficiency was evaluated using the CareStart™ diagnostic test, and enzyme activity levels were measured through fluorescence spectrophotometry in deficient individuals. Chi squared and ANOVA tests were used to assess prevalence and mean enzyme activity differences, and logistic regression was used to identify risk factors associated to the deficiency.

**Results:**

G6PD deficiency prevalence estimates were lowest in the northern city of Pemba (8.3%) and among Emakhuwas and Shimakondes, and higher in the centre and southern regions of the country (16.8 and 14.6%, respectively), particularly among Elomwes and Xichanganas. G6PD deficiency was significantly more prevalent among male students than females (OR = 1.4, 95% CI 1.0–1.8, p = 0.02), although enzyme activity levels were not different among deficient individuals from either gender group. Finally, median deficiency levels were found to be more severe among the deficient students from the north (0.7 U/gHg [0.2–0.7] p < 0.001) and south (0.7 U/gHg [0.5–2.5]), compared to those from the centre (1.4 U/gHg [0.6–2.1]).

**Conclusion:**

These findings suggest that Mozambique, as a historically high malaria-endemic country has considerable levels of G6PD deficiency, that vary significantly across the country. This should be considered when planning national strategies for the use of licensed drugs that may be associated to haemolysis among G6PD individuals, or prior to the performance of future trials using primaquine and other 8-aminoquinolines derivatives.

*Registration Number* CISM local ethics committee (CIBS-25/013, 4th of December 2013), and the National Ethics Committee of Mozambique (IRB00002657, 28th of February 2014).

## Background

Glucose-6-phosphate dehydrogenase (G6PD) deficiency is an X-linked hereditary enzymatic abnormality that affects more than 400 million people worldwide [1]. G6PD deficiency (G6PDd) has been historically linked to malaria endemic areas due to the believed protection that the deficiency offers against Plasmodium infections [2–4], specially among heterozygous females [5–7]. This deficiency is particularly prevalent in malaria endemic areas with the highest peaks in sub-Saharan Africa [8].

G6PD is a cytoplasmic enzyme expressed in all cells, involved in the first step of the pentose phosphate pathway of glycolysis. This enzyme is crucial for the protection of red blood cells from oxidative stress as it removes deleterious oxygen radicals, therefore avoiding premature erythrocytic lysis [9]. There are approximately 400 allele variants of the G6PD gene associated with different enzyme activity levels. The three most predominant variants in sub-Saharan Africa are (i) G6PD type B, correlated with a normal enzymatic activity; (ii) G6PD type A, coupled with an 85% enzymatic activity; and (iii) G6PD type A-associated with severe enzymatic activity of around 12% [10, 11]. Most deficient individuals do not manifest any symptoms; however, agents that cause excessive oxidative stress on human bodies can trigger acute haemolytic anaemia, which may often require lifesaving blood transfusions if severe [1], or other rarer manifestations of disease such as methaemoglobinaemia [12]. Precipitant factors include intake of fava beans, specific drugs, or infections [13, 14].

The most common chemicals associated with haemolysis among G6PD deficient individuals include anti-malarials from the 8-aminoquinoline family, such as primaquine, as well as other chemicals such as sulfonamides derivatives, dapsone or co-trimoxazole [1, 15, 16]. As a result, policies have been developed to avoid detrimental safety outcomes after the use of these drugs in settings where they are particularly necessary, and where the underlying population prevalence of G6PDd may be high. This is the case of primaquine (PQ), the currently only available 8-aminoquinoline drug, with the potential both to interrupt transmission given its efficacy against stage V gametocytes, necessary for malaria transmission, or to radically cure hypnozoites, responsible for *Plasmodium vivax* or *Plasmodium ovale* periodic relapses [14]. In this context, the World Health Organization (WHO) has recommended a low-dosage of primaquine coupled with an effective anti-malarial, for the purpose of interruption of malaria transmission at community level, that is safe for all individuals irrespective or a prior knowledge of their G6PD status [17]. However, doses recommended for the radical cure of *P. vivax* or *P. ovale* hypnozoites (7–14 days at higher doses than those recommended to interrupt transmission) are still associated with a high risk of haemolysis in individuals with severe enzymatic deficiency, and such treatment should not be administered before ascertaining G6PD status. Aside from primaquine, there are other 8-aminoquinoline derivatives in the anti-malarial pipeline, such as tafenoquine, which also pose similar challenges among G6PD deficient individuals [18]. In this context, countries should be informed of the prevalence of this enzymatic anomaly within their borders, in order to make safe and appropriate decisions regarding the use of potentially unsafe drugs for G6PD deficient individuals.

Information in Mozambique and the rest of sub-Saharan Africa regarding the frequency and activity level of G6PD deficiency is sparse. A study conducted in the northern province of Cabo Delgado in 1984 estimated an 18% prevalence of G6PD deficiency among males [19]. Studies conducted in Manhiça, Southern Mozambique, identified a proportion of G6PD type A-deficiency of 7.8 [11] and 10% [20] in a population of males and females, and a prevalence of 10% in children less than 5 years of age in a hospital-based study in Manhiça (Moraleda et al. pers. comm.). However, another study conducted in the city capital of Maputo, also in the south, found a 18% prevalence among male participants [21]. Given the topographical characteristics of Mozambique and its ethnic variability, the assessment of the frequency of G6PD deficiency in only one part of the country may not be representative of the national deficiency prevalence. The consequences from this heterogeneity become evident when observing differences in the distribution of other congenital disorders such as Hb S. In this case, evidence from published and unpublished studies identified very low Hb S carriers prevalent in the south [20] compared to the north of the country [19], which could be explained by ethnical heterogeneity. Although it is still unknown whether ethnical differences could have affected the distribution of G6PD deficiency in the same way, they need to be considered when measuring its prevalence.

This study aimed to assess the prevalence G6PD deficiency in three different and geographically distinct districts of Mozambique, quantify the levels of enzymatic activity in these populations, and identify risk factors associated to G6PDd.

## Methods

### Prevalence survey

**Fig. 1:**
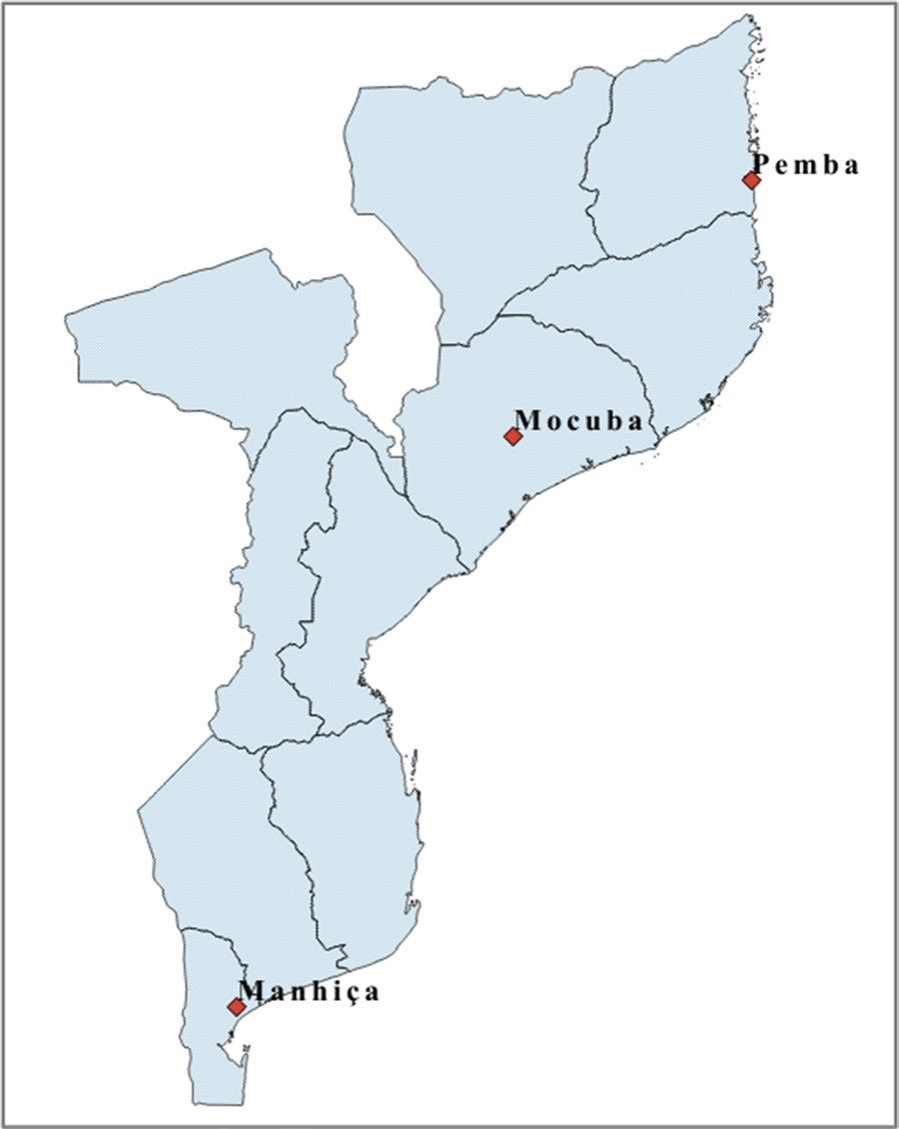
Map of study areas in Mozambique

A school-based cross-sectional survey was conducted in three districts in Mozambique, namely Manhiça, located in the south; Mocuba in the centre; and Pemba in the northern tip of the country (Fig. 1). Study sites were selected in order to evaluate the prevalence of G6PD in different regions of Mozambique and districts were selected based on logistical convenience. A sample size of 865 children was calculated per study area to estimate a 10% G6PDd prevalence with a precision of 2% and a confidence coefficient of 95%.

Two primary and two secondary schools were selected per study area. Students of both sexes aged 6 years or more attending the selected schools were approached and invited to participate in the study. For younger age students (6–17 year olds), parents/guardians were asked to provide a signed informed consent, and the students were also asked to provide their personal assent. Students 18 years of age or older were directly informed about the study, and requested to sign a written informed consent. All students who consented to participate in the study were included. Siblings or direct cousins of children already recruited were excluded from the study to avoid estimation bias due to consanguinity. A standardized questionnaire was completed for every study participant to collect basic demographic information. Ethnicity was assessed based on the mother tongue reported.

### G6PD deficiency estimates

Qualitative determination of G6PD deficiency was determined using the G6PDd CareStart™ rapid diagnostic test (RDT) according to the manufacturer instructions (AccessBio, New Jersey, USA). This is a qualitative enzyme chromatographic test, based on the reduction of colourless nitro blue tetrazolium dye to dark coloured formazan [22]. Two microlitres of blood were added into the sample well and two drops of buffer into the buffer well. Results were visually readable after 10–15 min. Samples with normal G6PD activity produced a distinct purple colour background in the result window, while no colour change indicated a deficiency in the G6PD activity. Deficiency levels detected by this test usually fall bellow 2.7 U/gHg of enzymatic activity.

Samples from subjects for whom the CareStart test showed a deficiency were used to quantify the residual enzyme activity (REA). Samples were processed at the Manhiça Health Research Centre (CISM) laboratory and conserved using the anticoagulant EDTA at 2–8 °C for a maximum of 5 days to guarantee the adequate preservation of red blood cells until determinations were performed.

Quantification of G6PD deficiency was measured using the Trinity Biotech quantitative G6PD assay™ (Ref. 345-UV, Trinity Biotech, St. Louis, USA), following the same methodology used to validate the performance of the CareStart test [23]. This method uses fluorescence spectrophotometry to analyze the rate of NADPH production from NADP by the G6PD enzyme from suspended-erythrocyte samples of 250 μL prepared using the readily available G6PDH assay and substrate reagents. G6PD activity was calculated from the increase in absorbance detected at 340 nm after 5 min of incubation corrected by the temperature of the reaction. The calculation also considered the haemoglobin concentration determined for each sample prior to spectrophotometry. The resulting G6PD activity was validated through a set of three controls provided by Trinity Biotech that were processed for each individual sample.

G6PD REA outcomes were categorized according to the WHO classification guidelines for G6PDd [1]: (i) Class I (very severely deficient (associated with chronic non-spherocytic haemolytic anaemia, <1% residual activity, <0.12 U/gHg), (ii) Class II (severely deficient, 1–10% residual activity, 0.13–1.2 U/gHg), (iii) Class III (moderately deficient, 10–60% residual activity, 1.3–7.1 U/gHg), (iv) Class IV (normal activity, 60–150% residual activity, 7.2–17.7 U/gHg) and (v) Class V (increased activity, >150% residual activity, >17.7 U/gHg) [23].

### Statistical analysis

Data were collected using Open Data Kit (ODK) software and managed using Stata 13.1 (Stata Corp., College Station, TX, USA). Proportions and 95% confidence intervals (CI) were calculated to estimate the prevalence of qualitative G6PDd individuals in males and females of each study area. Chi squared tests were used to assess a proportion difference in G6PDd individuals between gender groups for each area separately, and to assess the difference of the district-level G6PDd prevalence between areas.

Means and standard deviations of the REA spectrophotometry outcomes from G6PD deficient individuals’ were calculated to measure the severity of the deficiency in males and females of every district. Given the non-normal distribution of the REA measures obtained, Kruskal–Wallis tests were used to assess a difference in the median REA of deficient individuals between gender groups for each area separately, and of the district-level mean REA among areas.

Logistic regression was used to assess the level of association of each potential socio-demographic risk factor for RDT-based G6PD deficiency independently. All risk factors that were found to have a significant association (p value <0.05) to G6PD deficiency in the unadjusted model were included in a multi-variable model. To avoid multicollinearity caused by introducing area and ethnicity into the same model, two multivariate models were created, where the adjusted effect of area and ethnicity were separately estimated, controlling for the gender of participants. Effect modification parameters were introduced to assess the assumption of a combined association of variables that were believed a priori to have a correlated impact, such as the effect of the combination of the study area and the participant’s gender. However, the introduction of interaction parameters to assess this combined effect was not statistically relevant, and effect modification was finally excluded from the model. Likelihood ratios tests were used to (i) test for effect modification between the interaction variables, and (ii) assess the level of significance of the variables of the multivariate model, adjusted by all the other variables contained in the model.

## Results

Twelve schools, four per study area, were selected for this study, and a total of 2070 participants were recruited. Of these, 897 (43%) were from Pemba, 666 (32%) from Mocuba, and 507 (25%) from Manhiça. There was a slight overrepresentation of female participants in Pemba (57%) and in Manhiça (62%), and the mean age of participants was 17.9 (SD = 9.3) years old. The ethnicities reported were Emakhuwa (31%) and Shimakonde (6%), mostly present in Pemba; Elomwe (5%) in Mocuba; and Xichangana (21%) in Manhiça. A proportion of students in every study area belonged to other ethnicities, that overall represented 17% of the participants. These ethnic groups were not considered individually in the analysis due to their low representation in the overall sample (less than 5%). Additionally, a fifth (20%) of participants reported speaking only Portuguese, which limited our ability to assign them a particular ethnicity. Finally, a small sample of students (25) reported a history of blood transfusions (Table 1).

**Table 1:** Demographic characteristics of the participants per study area

	Pemba N (%)	Mocuba N (%)	Manhiça N (%)	Total N (%)
Gender				
Female	509 (57%)	254 (50%)	413 (62%)	1176 (57%)
Male	388 (43%)	253 (50%)	253 (38%)	894 (43%)
Age				
Mean (SD)	16.44 (7.84)	19.05 (10.24)	18.97 (10.18)	17.89 (9.33)
Transfusion				
No	892 (99%)	496 (98%)	653 (98%)	2041 (99%)
Yes	5 (1%)	8 (2%)	12 (2%)	25 (1%)
School				
1	210 (23%)	198 (39%)	176 (26%)	584 (28%)
2	159 (18%)	70 (14%)	69 (10%)	298 (14%)
3	349 (39%)	190 (37%)	304 (46%)	843 (41%)
4	179 (20%)	49 (10%)	117 (18%)	345 (17%)
Language^a^				
Emakhuwa	602 (67%)	35 (7%)	3 (0%)	640 (31%)
Shimakonde	126 (14%)	3 (1%)	0 (0%)	129 (6%)
Elomwe	2 (0%)	98 (19%)	3 (0%)	103 (5%)
Xichangana	12 (1%)	12 (2%)	408 (61%)	432 (21%)
Portuguese	28 (3%)	213 (42%)	172 (26%)	413 (20%)
Other	127 (14%)	146 (29%)	80 (12%)	353 (17%)

^a^Languages spoken by at least 5% of the study participants overall

**Fig. 2:**
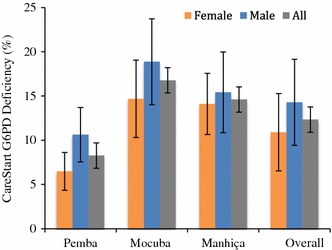
G6PD deficiency prevalence measured using the CareStart™ rapid diagnostic test by gender and study area

Of the 2070 individuals recruited, 2026 participants consented to be tested using the CareStart™ G6PD deficiency rapid diagnostic test. This analysis found significant differences (χ^2^ test p < 0.001) between each study area’s deficiency prevalence, estimated to be 8.3% (95% CI 6.5–10.1) in Pemba, 16.8% (95% CI 13.5–20.1) in Mocuba, and 14.6% (95% CI 11.8–17.1) in Manhiça. The stratification of G6PDd prevalence by gender in each study area revealed that 6.5% (95% CI 4.3–9.1) of female students from Pemba were deficient while the prevalence of G6PDd in males was significantly higher (10.6%, 95% CI 7.5–14.1, p = 0.03). Female students from Mocuba and Manhiça also showed lower G6PDd prevalence estimates (14.7 and 14.1% respectively) compared to their male counterparts (18.9 and 15.4% respectively), although these differences were not found to be statistically different (Table 2; Fig. 2).

**Table 2:** G6PD deficiency measured by the rapid diagnostic test CareStart™, and reactive G6PD enzyme activity of samples collected from RDT deficient individuals, measured by fluorescence spectrophotometry

	CareStart G6PD deficiency				Reactive G6PD activity^a^		
	Total	Deficient	% (95% CI)	χ^2^ test p value	Total	Median U/gHg (range)	Kruskal–Wallis p value
Pemba							
Females	509	33	6.5 (4.3–9.1)	0.03*	31	0.7 (0.2–0.7)	0.08*
Males	386	41	10.6 (7.5–14.1)		40	0.7 (0.2–0.7)	
All	895	74	8.3 (6.5–10.1)		71	0.7 (0.2–0.7)	
Mocuba							
Females	252	37	14.7 (10.3–19.1)	0.21*	33	1.4 (0.6–2.1)	0.08*
Males	249	47	18.9 (14.0–24.1)		40	2.1 (0.6–2.1)	
All	501	84	16.8 (13.5–20.1)		73	1.4 (0.6–2.1)	
Manhiça							
Females	390	55	14.1 (10.6–18.1)	0.65*	53	0.7 (0.5–2.5)	0.03*
Males	240	37	15.4 (10.8–20.1)		36	0.6 (0.5–2.5)	
All	630	92	14.6 (11.8–17.1)		89	0.7 (0.5–2.5)	
Total							
Females	1151	125	10.9 (9.1–13.1)	0.02^δ^	117	0.7 (0.2–2.5)	0.57^δ^
Males	875	125	14.3 (12.0–17.1)		116	0.7 (0.2–2.5)	
All	2026	250	12.3 (10.9–14.1)	<0.001^δ^	233	0.7 (0.2–2.5)	<0.001^δ^

* Chi Square or Kruskal–Wallis tests performed to assess the association between gender and G6PD Deficiency for each Area separately

^δ^Chi Square or Kruskal–Wallis tests performed to assess the association between study area or gender and G6PD Deficiency Overall

^a^REA assessed only for samples collected form G6PD Deficient Individuals as determined by the CareStart RDT

Unadjusted logistic regressions identified gender, study area and language (as a proxy for ethnicity) as significant risk factors for G6PDd diagnosed through CareStart™ RDT (LRT p < 0.05). Having a history of blood transfusions was not associated to G6PDd (OR = 0.7, 95% CI 0.2–2.9) and was, therefore, not considered in the multivariate model. Multivariate analysis revealed that males were 1.4 (95% CI 1.0–1.8) times more likely to be G6PD deficient compared to females. G6PD deficiency was significantly higher in Mocuba (OR = 2.2, 95% CI 1.6–3.1) and in Manhiça (OR = 1.9, 95% CI 1.4–2.7) compared to Pemba. Similarly, ethnic groups mainly prevalent in Mocuba (Elomwe) and in Manhiça (Xichangana) were 2.1 times more likely to be G6PD deficient compared to the Emakhuwa group from Pemba (Table 3).

**Table 3:** Univariate and multivariate logistic regression coefficients for the identification of risk factors associated to G6PD deficiency measured by RDT

Risk factors		G6PD deficiency		Unadjusted		Adjusted^a^		
		Prevalence (total)	95% CI	OR	95% CI	OR	95% CI	LRT p value
Gender	Female	0.11 (1151)	0.09–0.13	1		1		0.02
	Male	0.14 (875)	0.12–0.17	1.4	1.0–1.8	1.4	1.0–1.8	
Area	Pemba	0.08 (895)	0.07–0.10	1		1		<0.001
	Mocuba	0.17 (501)	0.14–0.20	2.2	1.6–3.1	2.2	1.6–3.1	
	Manhiça	0.15 (630)	0.12–0.18	1.9	1.4–2.6	1.9	1.4–2.7	
Language	Emakhuwa	0.09 (638)	0.07–0.12	1		1		0.002
	Shimakonde	0.09 (129)	0.05–0.16	1.0	0.5–2.0	1.0	0.5–1.9	
	Elomwe	0.17 (103)	0.11–0.26	2.1	1.2–3.8	2.1	1.2–3.7	
	Xichangana	0.17 (415)	0.14–0.21	2.1	1.4–3.0	2.1	0.5–1.9	
	Portuguese	0.12 (407)	0.09–0.16	1.4	0.9–2.0	1.4	1.2–3.7	
	Other	0.13 (334)	0.09–0.17	1.4	0.9–2.2	1.0	0.9–2.2	
Transfusion history	No	0.12 (1999)	0.11–0.14	1				
	Yes	0.09 (23)	0.01–0.28	0.7	0.2–2.9			

^a^Model adjusted for the variable gender

Samples collected from G6PD deficient individuals diagnosed using an RDT were analyzed to evaluate the degree of enzyme deficiency. Overall, G6PDd individuals from Pemba suffered from severe deficiency (Class II according to WHO classification), with a median G6PD enzyme activity of 0.7 U/gHg (0.2–0.7 U/gHg), corresponding to 1–10% residual activity. Median enzyme activity levels from G6PDd students in Mocuba and Manhiça were 1.4 U/gHg (0.6–2.5 U/gHg) and 0.7 U/gHg (0.5–2.5 U/gHg) respectively, corresponding overall to a moderate deficiency category (10–60% residual activity) according to WHO guidelines (Class III) in Mocuba and a severe deficiency in Manhiça. Nevertheless, REA levels ranged from Class II to Class III in both regions. Kruskal–Wallis tests revealed no significant differences in mean G6PD activities between males and females overall (Table 2).

## Discussion

This study sheds light over the current prevalence and intensity of G6PD deficiency in different regions of Mozambique. Results obtained from schools in the northern, middle and southern regions of the country suggest that G6PD prevalence varies significantly within the country, with the highest prevalence observed in the middle (Mocuba, 16.8%), followed by a slightly lower prevalence in the south (Manhiça, 14.6%), and the lowest prevalence in the north (Pemba, 8.3%).

As expected in a country with endemic malaria, G6PD deficiency prevalence estimates resemble those found in malaria endemic countries [4, 5, 24]. Mocuba, the area that showed the highest G6PDd prevalence in this study, belongs to the province of Zambezia, a high malaria-endemic region currently considered one of the world’s malaria hotspots, with recent malaria prevalence estimates at the community level ranging from 66.4% in 2007 [25] to 54.8% in 2011 [26]. However, G6PDd prevalence estimates found in the northern city of Pemba, also a malaria hyper-endemic region, are half the magnitude of the estimated prevalence in Mocuba, but appear significantly different to previous estimates in the area. A study performed in Pemba in 1986 showed a G6PDd prevalence of 18% among a male study population [19], which differs substantially from that found in this study among the male students of the same area (10.6%). This difference could be attributed to a difference in the sampling frame used to estimate prevalence, or merely due to chance. The population of Manhiça showed a G6PDd prevalence half way between the estimates that were previously found in the area (7.8 or 10%) and those measured in the neighbouring city of Maputo (18%) [21]. This confirms that G6PD prevalence is highly population specific, and thus, attention should be paid to the study population from which future estimates may be withdrawn.

The spatial heterogeneity of G6PD deficiency identified here could be partially explained by the ethnical differences existing within the country. However, as the association found between G6PD deficiency and ethnic group was highly correlated to the study site, this analysis was unable to make any inference about the effect of ethnicity independent from the region of the country where the majority of the ethnic groups are found and vice versa. This assessment was also limited by two additional factors. First, the schools selected for each region belonged to relatively urban areas, with higher probability of ethnical mixtures and a potential dilution of the association between reported ethnicity and enzyme deficiency. Second, a significant proportion of the study population reported Portuguese as their mother tongue, which hindered the capacity to identify the ethnic group to whom those participants belonged. Nevertheless, it is also possible that the spatial heterogeneity of the deficiency prevalence observed was merely due to chance, considering particularly the overlap of confidence interval estimates between Mocuba and Manhiça, which are substantially far away from each other.

Difference in G6PD deficiency rates between gender groups were only evident in Pemba, but were not significant in the middle and southern regions of the country, where the deficiency prevalence overall was also found to be higher. These findings correlate with the assumption that as the prevalence of G6PD deficient individuals increases in the population, so does the probability of finding homozygous female carriers of the deficient gene, who will express the deficiency in a more evident form [1]. However, regardless of the spatial differences in the association between gender and G6PD deficiency, the statistical modeling from this analysis identified no significant interaction between these two variables, suggesting that gender is still independently associated to the deficiency of the G6PD enzyme regardless of the population prevalence.

Additionally, this study did not find significant differences in enzymatic activity estimates between G6PD deficient males and females identified through the CareStart test. This is an unsurprising finding considering that the test excludes individuals with REA <2.7 U/g Hb) [23], which will predominantly include homozygous deficient females and hemizygous deficient males possibly with similar REA levels, This is particularly relevant in a context when community testing is required to identify severe deficiencies [27], as one can therefore assume that all severe forms of the deficiency will be detected regardless of the participant’s gender. The study further identified that deficiency levels in the north and south were significantly more severe to those in the centre of the country. Although it may be a stochastic finding, this difference could also be due to the presence of different G6PD gene variants across the country, which could confer varying enzyme activity levels among the deficient population [10]. However, this study did not conduct molecular analysis to identify the variant responsible for the observed deficiencies, which limited the capacity to interpret the observed regional patterns of enzyme activity levels. In addition, samples for enzyme activity determination were only collected for CareStart deficient individuals, which possibly limited our capacity to detect moderate deficiencies as well as to ensure that all severely deficient individuals were been identified by the test.

The results from this study are also subject to other limitations. First, the presence of punctual infections that cause haemolysis could have affected G6PD activity levels [13] in study participants who may otherwise not be identified as deficient. However, this analysis was not able to account for participants’ health status, which could lead to an overestimation of the prevalence of G6PDd individuals if a sample of participants with normal enzyme activity were suffering from haemolysis-related infections. Second, the study population among the three study sites was not homogenously distributed, as the northern region contributed to a larger proportion of the participants. Nevertheless, this did not affect the analysis, given that the estimates were always calculated per study area.

## Conclusion

This study shows a difference in G6PD deficiency prevalence within a sample of Mozambican students from three separate regions of the country. Prevalence estimates were lowest in the northern city of Pemba (8.3%) and among Emakhuwas and Shimakondes, and higher in the centre and southern regions of the country (16.8 and 14.6% respectively), particularly among Elomwes and Xichanganas. G6PD deficiency was significantly more prevalent among male students than females, although enzyme activity levels were not different among deficient individuals from either gender group. Finally, deficiency levels were found to be more severe among the G6PD deficient students from the north, compared to those from the rest of the country. These findings suggest that Mozambique, as a historically high malaria-endemic country, has considerable levels of G6PD deficiency across the country. This should be considered when planning national strategies for the use of licensed drugs that may be associated to haemolysis among G6PD individuals, or prior to the performance of future trials using primaquine and other 8-aminoquinolines derivatives.

## Abbreviations

ANOVA: analysis of variance
CI: confidence interval
G6PD: glucose-6-phosphate-dehydrogenase
G6PDd: glucose-6-phosphate-dehydrogenase deficiency
NADP: nicotinamide adenine dinucleotide phosphate
PQ: primaquine
RDT: rapid diagnostic test
REA: reactive enzyme activity
SD: standard deviation
WHO: World Health Organization
